# Recent Advances in Polymeric Membranes—Preparation and Applications

**DOI:** 10.3390/membranes16010003

**Published:** 2025-12-22

**Authors:** Maria Ortencia González-Díaz, Manuel Aguilar-Vega

**Affiliations:** 1 Centro de Investigación Científica de Yucatán, A.C., Calle 43 No. 130, Chuburná de Hidalgo, Mérida C.P. 97200, Yucatán, Mexico; 2 Secretaría de Ciencia, Humanidades, Tecnología e Innovación (SECIHTI), Av. Insurgentes Sur 1582, Col. Crédito Constructor, Demarcación Territorial, Benito Juárez C.P. 03940, Ciudad de Mexico, Mexico

## 1. Introduction

Polymeric membranes have gained increasing importance due to their low energy consumption, ease of operation, and favorable chemical, mechanical, and thermal stability [1]. Their versatility and relatively low cost have positioned them as essential materials in modern separation and purification technologies, motivating continuous innovation to meet increasingly demanding environmental, industrial, and biomedical applications, among others.

Across these diverse fields, recent research has converged on improving membrane robustness, selectivity, and long-term stability, together with advancing more sustainable and energy-efficient fabrication practices. Current efforts include enhancing resistance to fouling, extreme pH, oxidizing agents, and high-pressure operation, as well as incorporating greener solvents and environmentally conscious processing routes [2]. Moreover, there is growing interest in membranes capable of maintaining stable, energy-efficient performance during extended operation, particularly under variable feed conditions and frequent cleaning cycles [3]. Advances in polymer architecture, surface functionalization, and controlled polymerization have expanded the ability to tune membrane porosity, transport behavior, and biocompatibility. These strategies support current progress in water treatment and energy conversion systems, where optimized ionic transport and electrochemical stability are increasingly required, and biomedical technologies [4,5]. In particular, the capacity to engineer biomimetic morphologies allows polymeric membranes to approximate key characteristics of the extracellular matrix (ECM), enabling controlled drug delivery, improved cell–material interactions, and enhanced tissue regeneration.

The development of new polymeric materials and their growing number of applications necessitates the development of better-suited membranes with different characteristics or new composite membranes and methods of preparation, capable of meeting increasingly complex industrial, environmental, and medical demands. Their preparation can be achieved through various techniques and configurations, and ongoing research is continuously broadening the scope of materials and functionalities available. The following Special Issue, *Recent Advances in Polymeric Membranes—Preparation and Applications*, presents eight contributions, including both original research and reviews, that highlight recent progress across multiple fields.

## 2. Overview of Special Issue Contributions

In the field of water treatment and environmental remediation, the contributions addressed both sustainability and performance improvements in polymeric membranes. The authors of one study explored the revalorization of recycled high-impact polystyrene (HIPS) into asymmetric membranes for wastewater purification (Contribution 1). Through chemical sulfonation, sulfonic acid groups were successfully incorporated into the polymer structure, significantly improving surface hydrophilicity (with the contact angle decreasing from 83.8° to 66.1°) and enhancing antifouling resistance. As a result, the sulfonated membranes exhibited a marked increase in hydrophilicity and antifouling resistance, leading to a higher flux recovery ratio (FRR) compared with their non-sulfonated counterparts. The R-HIPS-5 membrane also achieved high rejection rates for Reactive Black 5 dye (94%) and divalent salts such as MgSO_4_ (72%) and Na_2_SO_4_ (67%), demonstrating that recycled plastics can be transformed into high-performance functional materials for sustainable water treatment. In another study (Contribution 2), the authors evaluated the long-term durability of poly(vinylidene fluoride) (PVDF) ultrafiltration membranes under repeated alkaline cleaning over a period of two years in a pilot-scale setup. Despite progressive pore enlargement and an over 50% increase in permeate flux, the quality of the treated water was preserved due to the formation of a thin fouling-induced separation layer that maintained high rejection of chemical oxygen demand (COD), turbidity, and surfactants. This finding confirms that PVDF membranes possess strong chemical resilience and can sustain efficient operation under industrial cleaning regimes.

Environmental challenges were also addressed in two comprehensive review papers. The authors of the first study (Contribution 3) focused on recent developments in polymer inclusion membranes (PIMs), highlighting advances in selectivity through the use of ionic liquids, task-specific carriers, and polymer functionalization techniques. They also addressed improvements in structural stability achieved with high-performance polymers and cross-linking strategies. Particular attention was paid to the shift toward sustainable fabrication, including the adoption of biodegradable polymers, green solvents, and scalable techniques such as phase inversion and electrospinning. These efforts collectively point to a new generation of PIMs that combine high separation efficiency, mechanical durability, and reduced environmental impact. In the second review (Contribution 4), the authors analyzed the performance of pressure-driven membrane processes, microfiltration (MF), ultrafiltration (UF), nanofiltration (NF), and reverse osmosis (RO), for removing micro- and nanoplastics from diverse aqueous matrices, including wastewater, surface water, and landfill leachate. Reported removal efficiencies reached up to 100%, depending on membrane type, pore size, and hydrodynamic conditions. The authors also discussed how membrane fouling, plastic particle aggregation, and the lack of standardized testing protocols remain major barriers to large-scale implementation. The authors emphasized that future progress will rely on improving fouling control strategies, optimizing membrane surface properties, and integrating membrane systems with complementary technologies such as coagulation or adsorption to enhance process robustness and scalability. Both reviews highlight how polymeric membranes are evolving to meet environmental demands while aligning with principles of sustainability and the circular economy.

In the field of energy applications, the authors of another included article (Contribution 5) focused on quaternized polysulfone membranes as anion-exchange solid polymer electrolytes for zinc–air batteries. This work contributes to the growing interest in polymer-based electrolytes as safer and more stable alternatives to conventional aqueous systems. By optimizing the degree of functionalization, the membranes achieved a hydroxide ionic conductivity of 22.19 mS·cm^−1^ and a peak power density of 70 mW·cm^−2^, both of which surpassed those of the commercial benchmark membrane (Fumapem FAA-3-50). In addition, postmortem analysis revealed reduced zinc oxide formation on the electrode surface, indicating enhanced rechargeability and lower passivation during cycling. These results highlight the critical role of polymer chemistry in advancing energy-conversion and storage technologies and demonstrate the potential of solid-state membranes to enable efficient, durable, and environmentally friendly electrochemical devices.

Regarding biomedical applications, the contributions reflected the increasing importance of polymeric membranes in health-related technologies. Electrospun polyacrylonitrile (PAN) membranes incorporating melanin derived from pecan nutshell residues exhibited strong antioxidant, antimicrobial, and enzyme-inhibiting properties, confirming the effectiveness of natural pigments as active fillers in polymer matrices (Contribution 6). The membranes containing up to 5 wt% melanin showed radical scavenging rates exceeding 80% for ABTS and 60% for DPPH assays, demonstrating a marked improvement compared to unmodified PAN fibers. In addition, they inhibited key skin-aging enzymes such as collagenase, tyrosinase, and elastase by up to 37%, 36%, and 33%, respectively, and displayed antibacterial activity against common pathogenic strains. These multifunctional features suggest potential use in skin-care and regenerative applications, illustrating how natural bioactive compounds can be successfully integrated into synthetic membranes to produce sustainable, value-added biomaterials. In another study (Contribution 7), the authors optimized field-emission scanning electron microscopy (FE-SEM) conditions to visualize the delicate porous structure of polypropylene membranes used in extracorporeal membrane oxygenation (ECMO) devices, enabling high-resolution imaging while minimizing morphological damage. The authors of another contribution reviewed the development pathway of a new hydrophilic polysulfone dialyzer, including its design, in vitro characterization, and clinical evaluation (Contribution 8). The modified membrane demonstrated reduced protein adsorption, improved hemocompatibility, and stable performance, illustrating how innovations in surface chemistry can translate into safer and more effective dialysis treatments.

## 3. Conclusions

Together, the contributions included in this Special Issue demonstrate how polymeric membranes are being advanced to address pressing global challenges. From water purification and environmental remediation to next-generation batteries and biomedical applications, these studies highlight the versatility of polymeric membranes and the continuing innovation in their preparation and application. Despite significant progress, challenges remain in scaling up sustainable fabrication methods, improving long-term stability, and translating laboratory developments into industrial and clinical contexts. We would like to thank all of the authors and reviewers for their valuable contributions, and we hope that this Special Issue will continue to inspire further research and innovation in the field of polymeric membranes.
